# Functional protection against cardiac diseases depends on ATP‐sensitive potassium channels

**DOI:** 10.1111/jcmm.13893

**Published:** 2018-09-14

**Authors:** Peng Ye, Yan‐Rong Zhu, Yue Gu, Dai‐Min Zhang, Shao‐Liang Chen

**Affiliations:** ^1^ Department of Cardiology Nanjing First Hospital Nanjing Medical University Jiangsu China

**Keywords:** cardiomyocytes, cardiovascular diseases, hydrogen sulphide, K_ATP_ channels, nitric oxide

## Abstract

ATP‐sensitive potassium channels (K_ATP_) channels are widely distributed in various tissues, including pancreatic beta cells, muscle tissue and brain tissue. K_ATP_ channels play an important role in cardioprotection in physiological/pathological situations. K_ATP_ channels are inhibited by an increase in the intracellular ATP concentration and are stimulated by an increase in the intracellular MgADP concentration. Activation of K_ATP_ channels decreases ischaemia/reperfusion injury, protects cardiomyocytes from heart failure, and reduces the occurrence of arrhythmias. K_ATP_ channels are involved in various signalling pathways, and their participation in protective processes is regulated by endogenous signalling molecules, such as nitric oxide and hydrogen sulphide. K_ATP_ channels may act as a new drug target to fight against cardiovascular disease in the development of related drugs in the future. This review highlights the potential mechanisms correlated with the protective role of K_ATP_ channels and their therapeutic value in cardiovascular diseases.

## 1 INTRODUCTION

Arrhythmia is a leading cause of mortality and morbidity in cardiovascular diseases. Several ion channels in cardiomyocytes shape action potentials, trigger electrophysiological activities, and excitation‐contraction coupling (ECC), and finally, induce cardiac contraction and blood pumping within the circulatory system. Abnormal ion channels may affect cardiac function.

The ATP‐sensitive potassium (K_ATP_) channel was first identified by Noma in cardiomyocytes treated with hypoxia using the patch clamp technique in 1983.^1^ K_ATP_ channels are characterized by channel inhibition because of an increase in the intracellular ATP concentration and stimulation because of an increase in the intracellular MgADP concentration.^2^, ^3^, ^4^ Cumulative evidence has indicated that K_ATP_ channels, which are ATP‐sensitive potassium channels, are widely distributed in various tissues, including pancreatic beta cells, muscle tissue, and brain tissue, and play an important role in cardioprotection in physiological and/or pathological states. Particularly, under pathophysiological conditions, K_ATP_ channels play a critical protective role by regulating cardiac repolarization. Activation of K_ATP_ channels decreases ischaemia/reperfusion injury, protects cardiomyocytes during heart failure, and reduces the occurrence of arrhythmias.

K_ATP_ channels couple the cellular metabolic state to the membrane potential. K_ATP_ channels play a critical role in various cellular functions, including hormone secretion and regulation of muscle excitability. K_ATP_ channels are also the target of endogenous vasoactive substances, such as nitric oxide (NO), reactive oxygen species (ROS), and hydrogen sulphide (H_2_S).^5^, ^6^ The protective mechanisms of K_ATP_ channels involve various signalling pathways. K_ATP_ channels may act as a new drug target to fight against cardiovascular disease in the development of drugs in the future. This review highlights a potential mechanism correlated with the protective role of K_ATP_ channels and their therapeutic value in cardiovascular diseases.

## 2 PROPERTIES OF K_ATP_ CHANNELS

**Figure 1:**
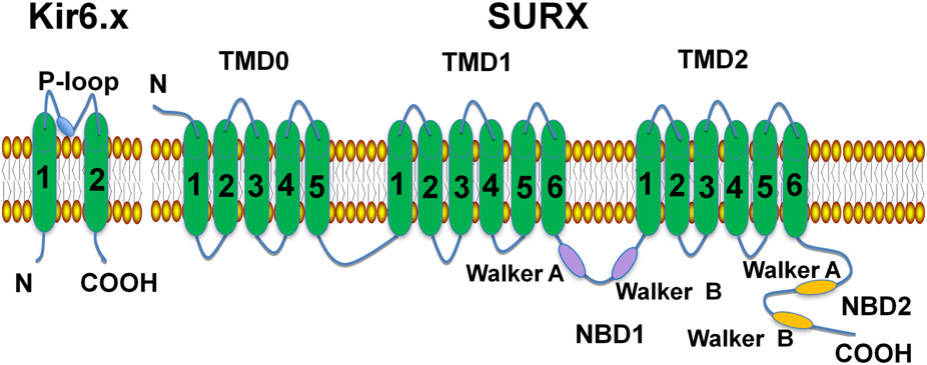
Schematic structure of an ATP‐sensitive potassium channel. The K_ATP_ channel consists of Kir6.x (Kir6.2 and Kir6.1) and the regulatory subunits of SURx (SUR1, SUR2A, and SUR2B). The Kir6.x subunit has two transmembrane regions with intracellular NH_2_ and COOH termini. The SURx subunit has 17 transmembrane regions, which include three domains: TMD0, TMD1, TMD2. SURx has two conserved intracellular nucleotide binding domains (NBDs). NBD1 is located between TMD1 and TMD2, whereas NBD2 exists in the COOH terminal of TMD2

**Figure 2:**
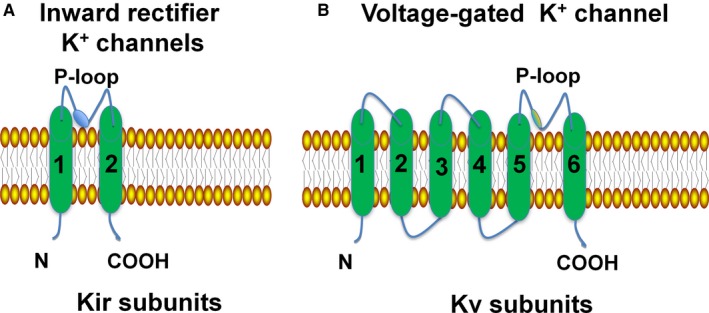
Structure of the Kir channel and Kv channel. SarcK_ATP_ channels are composed of eight proteins, which include four members of the inward rectifier K^+^ channel family Kir6.x, and four sulfonylurea receptors. The Kir subunit contains two transmembrane regions, a pore‐forming loop and cytosolic NH_2_ and COOH termini, whereas the Kv channel subunit possesses six transmembrane regions, which include two functionally and independent domains: an ion conduction pore, and voltage‐sensor domains. Kir channel: inwardly rectifying K^+^ channel; Kv channel: voltage‐gated K^+^ channel; sarcK_ATP_ channel: sarcolemmal ATP‐sensitive potassium channel

Many K^+^ channels are regulated by voltage and/or Ca^2+^ and are named Kv or KCa channels respectively.^7^, ^8^, ^9^ A group of K^+^ channels that is not regulated in this manner is the inward rectifier K^+^ channel (Kir channels). K_ATP_ channels are composed of four Kir channel subunits, Kir6.x (Kir6.1 and Kir6.2 are encoded by KCNJ8 and KCNJ11, respectively), and four sulphonylurea receptors, SUR (SUR1 and SUR2, with two splice variants: SUR2A and SUR2B, which are encoded by ABCC8 and ABCC9, respectively), whose subunit composition exhibits tissue specificity^10^, ^11^ (see Table 1). SUR has three domains that include TMD0, TMD1, and TMD2 helices. TMD1‐TMD2 and the C‐terminus contain nucleotide‐binding domains (NBD1 and NBD2)^12^, ^13^ (see Figure 1). The Kir subunit contains two transmembrane regions, a pore‐forming loop and cytosolic NH_2_ and COOH termini, whereas the Kv (voltage‐gated K^+^) channel subunit possesses six transmembrane regions, which include an ion conduction pore and voltage‐sensor domains^14^ (see Figure 2).

**Table 1:** Subunit genes of K_ATP_ channels

Isoforms	Gene	Chromosome
Kir6.2	KCNJ11	11p15.1
Kir6.1	KCNJ8	12p11.23
SUR1	ABCC8	11p15.1
SUR2	ABCC9	12p12.1

KCNJ11: potassium inwardly rectifying channel, subfamily J, Member 11; KCNJ8: potassium inwardly rectifying channel, subfamily J, Member 8; ABCC8: ATP‐binding cassette, subfamily C, member 8; ABCC9: ATP‐binding cassette, subfamily C, member 9.

Kir6.2/SUR2A is present in ventricular muscle cells, and Kir6.1/SUR2B is present in smooth muscle cells. The Kir6.2 and SUR1 subunits are present in pancreatic beta cells and neurons of the central nervous system^15^ (see Table 2). The Kir6.2 subunit has more intrinsic sensitivity to cellular metabolic disorders than the Kir6.1 subunit.^16^ In addition, the composition of the mitochondrial K_ATP_ (mitoK_ATP_) channels is still unclear, although some literature suggests that Kir6.1 may be a functionally important part of mitoK_ATP_ channels in native heart cells.^17^

**Table 2:** Diverse properties of K_ATP_ channels in cardiovascular tissue

Tissue	Conductance (pS)	Subunit composition
Atrium	52‐85	Kir6.2/SUR1/SUR2A
Ventricle	75‐85	Kir6.2/SUR2A
Conduction System	52‐60	Kir6.2/Kir6.1/SUR2B
Mitochondria	15‐100	Kir1.1/SUR2A

## 3 CARDIAC DISEASES

The opening and closing of K_ATP_ channels are regulated by various signalling molecules, including membrane phosphoinositides, the intracellular ATP concentration, and MgADP.^18^ Small signalling molecules, such as NO, H_2_S, and ROS, also play critical roles in protecting the cardiovascular system by regulating K_ATP_ channels.^5^, ^6^ H_2_S is endogenously generated from cysteine metabolism. Zhao et al^6^ reported that H_2_S directly increased K_ATP_ channel currents and hyperpolarized membrane and relaxed rat aortic tissues *in vitro* in a K_ATP_ channel‐dependent manner. These results demonstrated that H_2_S is an important endogenous vasoactive factor and a gaseous opener of K_ATP_ channels in vascular smooth muscle cells, but their mechanisms of action remain unclear. Zhang et al^5^ found that NO, a gaseous messenger known to be cytoprotective, increased the activity of K_ATP_ channels. These changes were reversed in the presence of inhibitors that were selective for PKG, ERK, calmodulin, or genetic ablation of CaMKIIδ, the predominant cardiac CaMKII isoform. These experimental results indicate that NO modulates K_ATP_ channels via a novel PKG‐ERK‐calmodulin‐CaMKIIδ signalling pathway.^5^

### 3.1 Cardiomyopathy

The opening of K_ATP_ channels protects cardiac muscle cells against hyperglycaemia‐induced damage and inflammation by inhibiting the ROS‐activated TLR4‐necroptosis pathway, which may be an important underlying mechanism of ROS in diabetic cardiomyopathy. These results indicated the cardio‐protective effects again injury induced by ROS in a KATP channels‐dependent manner.^19^

Some binding sites in K_ATP_ channels combine with signalling molecules to regulate the activity of K_ATP_ channels. Burton et al^20^ found that cardiac K_ATP_ channels were regulated by heme, and the cytoplasmic heme‐binding CXXHX16H motif on the SUR subunit of the channel was shown to include Cys628 and His648, which are important for heme binding. These results supported the hypothesis that there are mechanisms of heme‐dependent regulation across other ion channels.

Activation of α_1_‐adrenoceptors by pretreatment with phenylephrine can up‐regulate SUR2B/Kir6.2 to participate in cardio‐protection in H9C2 cells.^21^ This study showed that the up‐regulation of SUR2B/Kir6.2 might have a different physiological consequence from the up‐regulation of SUR2A/Kir6.2. This is the first explanation for the possible physiological role of SUR2B in a cardiac phenotype.

Syntaxin‐1A interacts with SUR2A to inhibit K_ATP_ channels, and PIP_2_ is known to bind the Kir6.2 subunit to open K_ATP_ channels. By contrast, it is interesting that PIP2 affects K_ATP_ channels by dynamically modulating Syn‐1A mobility from Syn‐1A clusters, resulting in the availability of Syn‐1A to inhibit K_ATP_ channels on the plasma membrane. This differential effect is related to the concentration of PIP_2._ 22

Evidence demonstrates that stretch‐induced K_ATP_ channel activity is controlled by MgATPase activity. These results may explain how K_ATP_ channel activity might respond rapidly to changes in the cardiac workload in a healthy heart and in a pathological state.^23^ In addition, phosphorylating NBD1 of SUR2B also exposes the MgATP binding site, thus disrupting the interaction of the NBD core with the N‐terminal tail and, in turn, activating K_ATP_ channels.^24^

### 3.2 Cardiac arrhythmias

Cardiac K_ATP_ channels have emerged as crucial controllers of heart failure and ischaemia‐related cardiac arrhythmias and have been suggested to be responsible for cardioprotection by decreasing repolarization dispersion and promoting action potential shortening.^25^, ^26^, ^27^

In cardiomyocytes, the ability of the mitochondrial network, which restores energy production and limits necrotic and apoptotic cell death, is a key determinant of survival after ischaemia‐reperfusion (IR). Mitochondrial function is a major factor in arrhythmogenesis during IR. Mitochondrial uncoupling can alter cellular electrical excitability and increase the propensity for reentry through opening of sarcolemmal K_ATP_ channels. Mitochondrial inner membrane potential (ΔΨm) is an essential component in the process of energy storage. ΔΨm instability or oscillation induced by ROS led to cardiac arrhythmia. This mechanism is necessary to increase the dispersion of refractoriness, slow the conduction velocity, and create a regional excitation block.^28^

Recent data have shown that mutations of K_ATP_ channels in myocardial cells can directly result in arrhythmias. The E23K variant of KCNJ11, identified in patients with type 2 diabetes,^29^ results in the frequent opening of K_ATP_ channels, which is associated with a greater left ventricular size with hypertension.^30^ These mutations, in turn, increases the occurrence of ventricular arrhythmias (VA) in dilated cardiomyopathy patients. Moreover, a KCNJ8 mutation was also found to be associated with atrial fibrillation (AF), and K_ATP_ channel currents were found to be decreased during chronic human AF.^31^

Feng et al^32^ reported that by over‐expressing the E23K variant, ventricular electrophysiological instability was increased when impaired by acute ischaemia. However, low‐doses of diazoxide, a K_ATP_ channel opener, improved hypoglycaemia‐related complications in patients with type 2 diabetes mellitus (T2DM), resulting in rapid and heterogeneous APD shortening to promote reentrant ventricular tachyarrhythmias during ischaemia.^33^

### 3.3 Mutations in human disease

Patients with Cantu syndrome, which is caused by gain‐of‐function (GOF) mutations in genes encoding Kir6.1 and SUR2, have enlarged hearts with an increased ejection fraction and increased contractility. Mice with cardiac‐specific Kir6.1 GOF subunit expression show left ventricular dilation and increased basal L‐type Ca^2+^ currents, which results in phosphorylation of the pore‐forming α_1_ subunit of the cardiac voltage‐gated calcium channel Cav1.2 at Ser1928 relative to that in WT mice treated with isoproterenol.^34^ This result suggests that increasing protein kinase activity may act as a potential connection between increased K_ATP_ current and cardioprotection.

## 4 DRUG THERAPY AND PERSPECTIVE

K_ATP_ channels, a major drug target for the treatment of T2DM, are crucial in the regulation of heart injury and vascular smooth muscle tone. K_ATP_ channels are also important since the therapeutic agents that target K_ATP_ channels can also treat cardiovascular diseases. New effects correlated with K_ATP_ channels have recently been identified that were previously undetected. For example, the volatile anaesthetic isoflurane preserves the normalization of human K_ATP_ channel activity in human arteries exposed to oxidative stress caused by high glucose.^35^ Isoquercitrin induces vasodilation in resistance arteries, an effect that is mediated by the opening of K_ATP_ channels and endothelial NO production.^36^

Treatment with one type of fatty acid, EPA, in the early phase of myocardial infarction significantly reduces cardiac mRNA and protein expression of Kir6.2 and increases the SUR2B subunit. These findings indicate that EPA may suppress acute phase fatal VA and may have an inhibitory effect on ischaemia‐induced ventricular fibrillation *in vivo*, which may be mediated by an enhancement of ischaemia‐induced monophasic action potential shortening.^37^

Epoxyeicosatrienoic acids, metabolites of arachidonic acid, are involved in the activation of PI3Kα and opening of K_ATP_ channels, which prevent Ca^2+^ overload and maintain mitochondrial function.^38^ Isosteviol sodium may increase the activation rate of sarcK_ATP_ channels induced by pinacidil and potentiate the diazoxide‐elicited oxidation of flavoproteins in mitochondria.^39^

The new antihypertensive drug iptakalim activates K_ATP_ channels in the endothelial cells of resistance blood vessels, a mechanism that is dependent on ATP hydrolysis and specific ATP ligands. The functions of endothelial K_ATP_ channels in resistance blood vessels can be changed by the exposure to the high shear stress caused by hypertension.^40^

Sulfonylurea induces the release of insulin by inhibiting the K_ATP_ channels that are present in pancreatic beta cells. Under high glucose conditions, glucose promotes the synthesis of intracellular ATP, which blocks K_ATP_ channels and prevents K^+^ efflux, leading to membrane depolarization and the opening of Ca^2+^ channels, allowing an influx of calcium and release of insulin.^41^, ^42^ Although efforts to reduce cardiovascular mortality in patients with diabetes mellitus should focus on improving glycaemic control, there is controversy as to whether the use of sulfonylurea increases the susceptibility of the myocardium to ischaemic insult, in light of the functional mechanisms of sulfonylurea drugs, which act through inhibition of the K_ATP_ channels present in both pancreatic beta cells and cardiomyocytes.^25^, ^26^, ^27^, ^43^, ^44^ Cardiac K_ATP_ channels inhibited by sulfonylurea drugs may be harmful to the ischaemic myocardium because of a disturbed K_ATP_ channel dependent response in the ischaemic precondition response.^26^ Further research is needed to develop a “selective” drug that targets pancreatic KATP channels without affecting cardiac channels.

It is clear that K_ATP_ channels play a significant role in the protection against cardiovascular diseases. In the literature, there is a considerable volume of information available regarding the mechanism of the protective role of K_ATP_ channels under different pathophysiological conditions, such as I/R injury, heart failure, cardiac arrhythmias, and atherosclerosis. However, studies characterizing K_ATP_ channels in targeted therapy of patients as well as the research and development of efficacious therapies are sparse, and further studies should investigate the potential mechanisms of K_ATP_ channels in cardiovascular diseases.

## CONFLICT OF INTEREST

The authors declare that they have no conflict of interest.
